# Multiparametric Magnetic Resonance Imaging-Ultrasound Fusion Transperineal Prostate Biopsy: Diagnostic Accuracy from a Single Center Retrospective Study

**DOI:** 10.3390/cancers13194833

**Published:** 2021-09-28

**Authors:** Andrea Fulco, Francesco Chiaradia, Luigi Ascalone, Vincenzo Andracchio, Antonio Greco, Manlio Cappa, Marcello Scarcia, Giuseppe Mario Ludovico, Vincenzo Pagliarulo, Camillo Palmieri, Stefano Alba

**Affiliations:** 1 Department of Urology, Romolo Hospital, 88821 Rocca di Neto, Italy; andreafulco.md@gmail.com (A.F.); francescochiaradia@romolohospital.com (F.C.); luigiascalone@romolohospital.com (L.A.); andracchio.v@libero.it (V.A.); antoniogreco@romolohospital.com (A.G.); manlio.cappa@gmail.com (M.C.); 2 Department of Clinical and Experimental Medicine, University Magna Grecia of Catanzaro, 88100 Catanzaro, Italy; cpalmieri@unicz.it; 3 Department of Urology, Ente Ecclesiastico Ospedale Generale Regionale “F. Miulli”, 70021 Acquaviva delle Fonti, Italy; m.scarcia@miulli.it (M.S.); g.ludovico@miulli.it (G.M.L.); 4 Department of Urology, Ospedale Vito Fazzi, 73100 Lecce, Italy; enzopagliarulo@yahoo.com

**Keywords:** image-guided, magnetic resonance imaging, ultrasonography, prostatic neoplasms, biopsy

## Abstract

**Simple Summary:**

The introduction of imaging techniques has improved the diagnostic pathway for prostate cancer. In this study we compared the diagnostic accuracy of multiparametric MRI with fusion ultrasound-guided prostate biopsy and standard biopsy, both performed through the transperineal route. Our results support the combined targeted and standard biopsy pathway to reduce the risk of missing clinically significant prostate cancer.

**Abstract:**

The management of prostate biopsy in men with clinical suspicion of prostate cancer has changed in the last few years, especially with the introduction of imaging techniques, to overcome the low efficacy of risk stratification based on PSA levels. Here, we aimed to compare the diagnostic accuracy of multiparametric MRI with fusion ultrasound-guided prostate biopsy and standard biopsy, both performed through the transperineal route. To this end, we retrospectively analyzed 272 patients who underwent combined transperineal targeted and standard biopsy during the same session. The primary outcome was to compare the cancer detection rate between targeted and standard biopsy. The secondary outcome was to evaluate the added value of combined targeted and standard biopsy approach as compared to only targeted or standard biopsy. Results showed that a rate of 16.7% clinically significant tumors (International Society of Urological Pathology (ISUP) grade ≥ 2) would have been lost if only the standard biopsy had been used. The combined targeted and standard biopsy showed an added value of 10.3% and 9.9% in reducing the risk of prostate cancer missing after targeted or standard biopsy alone, respectively. The combined targeted and standard biopsy pathway is recommended to reduce the risk of missing clinically significant prostate cancer.

## 1. Introduction

Prostate cancer (PCa) is a leading cause of cancer death in men. The diagnostic pathway of PCa in men presenting with symptoms referable to a possible prostate disease includes the combined use of digital rectal examination (DRE), serum biomarkers, imaging techniques, and biopsy. The introduction of robust prostate-specific antigen (PSA) assay has long fostered the possibility of screening for early disease prediction in asymptomatic men. In addition to PSA, other serum and urinary biomarkers (reviewed in [1]) have been identified and approved by the Food and Drug Administration (FDA) and Clinical Laboratory Improvement Amendments (CLIA) for improving PCa diagnosis and prognosis, and helping in biopsy decision. 

Over the past decade, the introduction of multiparametric magnetic resonance imaging (mpMRI) and mpMRI-Ultrasound Fusion-Targeted (TBx) has raised great expectations about the diagnostic pathway of PCa. The large-scale Patient-Reported Outcomes Measurement Information System (PROMIS) multicenter study indicated that mpMRI, when performed as a triage test prior to biopsy, has a two-fold advantage: on the one hand, it significantly reduces overdiagnosis and overtreatment of clinically indolent tumors; on the other hand, it significantly increases the diagnosis of clinically significant tumors compared to ultrasound (US)-guided random biopsy [2]. Many studies comparing the diagnostic accuracy between TBx and standard biopsy (SBx) in detecting clinically significant PCa (ISUP grade ≥ 2) demonstrated the superiority of TBx in the repeat-biopsy setting [3]. Even in biopsy-naïve patients, TBx out-performed SBx, but the difference appears to be less pronounced and insignificant [2,4]. Furthermore, TBx appeared to detect fewer patients with clinically insignificant PCa (ISUP grade 1, maximum core length < 6 mm) than SBx. In consequence, TBx was superior to SBx in reducing overdiagnosis of low-risk disease [3,4,5,6]. 

Many studies have evaluated the combined diagnostic pathway, in which SBx and TBx biopsy was performed in the same patients with a positive mpMRI. The data from the Cochrane meta-analysis of these studies indicated that the absolute added value of TBx for detecting ISUP grade > 2 cancers is higher than that of SBx [3].

Prostate lesions found on mpMRI are graded from 1 to 5, according to the Prostate Imaging-Reporting and Data System (PIRADS) version 2, where higher imaging suspicion scores are associated with a higher risk of clinically significant PCs (csPCa) [7]. A score of “3” indicates equivocal results, “4” results are likely to be prostate cancer, and “5” results are highly likely to be prostate cancer.

The TBx can be achieved through a transrectal or transperineal route. Both approaches are equivalent for patient tolerability and PCa detection rates in an SBx setting, with slight differences in infectious and retention complications [8,9,10]. However, the comparison in cancer detection rate (CDR) in a TBx configuration remains unclear.

The purpose of this study was to compare the CDR of TBx and SBx, both performed through the transperineal route. An additional outcome was to evaluate the added value of the combined TBx and SBx approach as compared to only TBx or SBx biopsy.

## 2. Materials and Methods

We analyzed data from a database of 272 patients who underwent primary or repeated prostate biopsy at a single institution in the period from December 2017 to February 2020. All patients gave their informed consent to use the data obtained from medical records; biopsies were performed according to standard procedures, and no additional biopsy was performed for this study; in addition, patient identification information was anonymized before analysis. All procedures performed in this study were in accordance with the Helsinki declaration and its later amendments for comparable ethical standards.

Data were from men aged at least 18 years, referred with a clinical suspicion of prostate cancer based on elevated levels of prostate-specific antigen (PSA, ≥4 ng/mL) and/or suspicious DRE results or family history of prostate cancer that were fit to undergo all protocol procedures, including a transrectal ultrasound. Patients were excluded if they used 5-alpha-reductase inhibitors during the previous six months, had a history of prostate cancer, or had evidence of urinary tract infection or acute prostatitis. All patients had a recent prostate-mpMRI (<45 days) with at least one lesion with PIRADS v2 score ≥ 3 (Study Flow Chart, Figure S1).

The mpMRI was performed with a 1.5-T MRI scanner using a 32-channel phased-array coil combined with an endorectal coil and included three orthogonal triplanar T2-weighted (T2w), diffusion-weighted imaging (DWI) with calculated *b*-value images, axial apparent diffusion coefficient (ADC) map, and dynamic contrast-enhanced (DCE) image sequences. All patients were scanned with the same MRI protocol, MRI scanner, and software version. Details of the MRI protocol are provided in Supplemental Materials. The MRIs were reviewed by two experienced uro-radiologists and scored according to the PIRADS v2.

Each participant underwent TBx and SBx during the same session, with TBx taking place prior to SBx. The same person performed both TBx and SBx. The number of biopsies on targeted areas of the mpMRI-US and random biopsies were performed according to guidelines [11]. For SBx, a total of 10 biopsy cores were obtained from the peripheral zone of the prostate at the base, mid gland, and apex. For TBx, three to five biopsy cores per target were obtained for participant; the transition zone was not subjected to routine biopsy, except in the case of positive mpMRI. All biopsies were performed using the transperineal approach under monitored anesthesia, or local anesthesia, by two operators with experience of more than 1000 procedures. Biopsies performed during the learning curve were excluded from the analysis. Fusion biopsies were carried out with the Koelis Trinity system (Koelis, Meylan, France). Koelis Trinity system creates a precise and highly detailed 3D map of the prostate, showing the biopsy cores locations and suspicious areas delineated on mpMRI sequences. Trinity integrates 3D ultrasound, multimodal elastic fusion, and Organ-Based Tracking, which allows the device to follow the prostate’s position and not that of the probe, automatically compensating for patient movement and prostate deformation.

All biopsy cores were analyzed by a centralized pathological anatomy laboratory and by a single operator with experience in uropathology and reported according to ISUP 2014 criteria [12,13]. Clinically significant (csPCa) was defined as having an ISUP score ≥ 2, and clinically insignificant (ciPCa) prostate cancer was defined as having an ISUP score < 1.

Descriptive statistics was evaluated using GraphPad Prism version 9.0.0 (GraphPad Software, San Diego, CA, USA). Difference of detection rates between TBx snd SBx was evaluated by McNemar’s test, Chi-square, and Fisher’ exact test. Statistical significance was established for *p* < 0.05. The added values were calculated by considering the cancer prevalence in the entire cohort.

## 3. Results

Our cohort consisted of 272 patients who had a mpMRI result suggestive of prostate cancer (PIRADS v2 score, ≥3), and underwent TBx and SBx during the investigated period. Eighty-two patients (30.1%) had at least one prior negative biopsy, whereas 190 (69.9%) were biopsy-naïve. Our cohort included 115 cases (42.3%) with areas of the prostate classified as PIRADS 3, 129 cases (47.4%) as PIRADS 4, and 28 cases (10.3%) as PIRADS 5. Our cohort consisted of 272 patients who had a mpMRI result suggestive of prostate cancer (PIRADS v2 score, ≥3), and underwent TBx and SBx during the investigated period. In total, 82 patients (30.1%) had at least one prior negative biopsy, whereas 190 (69.9%) were biopsy-naïve. Patients’ characteristics and PIRADS classification are summarized in Table 1.

**Figure 1:**
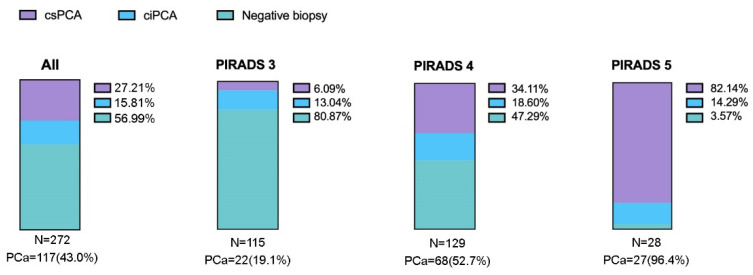
Percentages of patients with clinically significant (csPCa), insignificant (ciPCa), and no cancer in the whole cohort (leftmost bar chart) or according to PIRADS v2 score (remaining bar charts).

Overall, prostate cancer was detected in 117 of 272 men (43.0%), including 74 csPCa (27.2%) and 43 ciPCa (15.8%). The highest rate of csPCa was observed in PIRADS 5 index lesions, while the highest rate of negative biopsies was observed in PIRADS 3 (Figure 1).

PCa was detected more frequently in biopsy-naïve patients than in patients undergoing prior biopsy, while negative biopsies were more frequent in prior biopsy patients (*p* = 0.0162; Table 2). The amount of csPCa was also more frequent in biopsy-naïve patients than in patients undergoing previous biopsy (31.6% versus 17.1% of all PCa, *p* = 0.0136), the latter showing a comparable frequency of csPca and ciPCa (Table 2). There was no significant difference between TBx and SBx biopsy in the detection rate of any PCa (32.7% vs. 33.1%), csPCa (22.4% vs. 22.4.0%), ciPCa (10.3% vs. 10.7%), or negative biopsy (67.3% vs. 66.9%, *p* = 1), as assessed by McNemar’s test (Table 3).

Notably, we observed a comparable number of patients showing either TBx-negative and SBx-positive results, or SBx-negative and TBx-positive results (27 and 28, respectively, Table 3), indicating that the combined TBx and SBX biopsy has an added value of 10.3% and 9.9% in reducing the risk of PCa missing after TBx or SBx alone, respectively (examples of mpMRI image for each clinical scenario are provided as Supplemental Figures S2–S4). Furthermore, only 62/117 PCas (53.0%) were positive for both biopsy approaches, meaning that 12/43 ciPCa and 26/74 csPCa would not have been diagnosed if patients had undergone TBx alone, or SBx; these data demonstrate an added value of 10.3% and 9.6% of the combined biopsy in the detection of ciPCa and csPCA, respectively.

**Figure 2:**
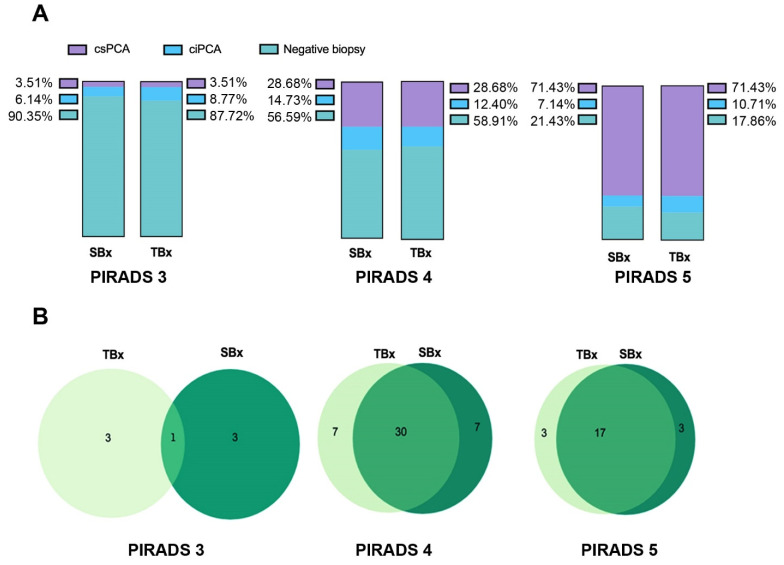
Diagnostic accuracy of TBx and SBx. (**A**) Percentages of patients with csPCa, ciPCa, or with negative biopsy revealed by TBx or SBx according to the PIRADS v2 score. (**B**) Venn diagrams showing the number of csPCa detected by TBx alone, SBx alone, or by the combined TBx/SBx approach.

The CDRs of TBx and SBx according to PIRADS subgroups were not significantly different (Figure 2A). For PIRADS 3 lesions, only one csPCa was detected by both TBx and SBx, while three out of seven csPCa were detected by TBx or SBx alone (Figure 2B).

Similarly, in PIRADS 4 and 5 lesions, a certain number of csPCa were detected either only after TBx, or only after SBx, although most of them were detected by both biopsy approaches. Overall, 13/74 (17.6%) of csPCa would not have been diagnosed if patients had undergone TBx alone, and 13/74 (17.6%) of csPCa would not have been diagnosed if patients had undergone SBx alone. These results further emphasize the non-redundant value for the two approaches in detecting csPCa. No major complications (Clavien Grade 3–4) were observed after 272 biopsies, but only minor complications that did not require hospitalization, such as hematospermia (38%), hematuria (8%), and fever > 38 °C (0.01%).

## 4. Discussion

The management of prostate biopsy in men with clinical suspicion of PCa has changed in the last 10 years, especially with the introduction of imaging techniques, in order to overcome the low efficacy of risk stratification based on PSA levels [5]. To implement proper treatment, extensive efforts have been made to identify csPCa. Several studies have reported the superiority of targeted and systematic biopsy based on mpMRI compared to US-guided transrectal biopsy [2,4,5]. The ideal detection method for diagnosing csPCa should be minimally invasive, have fewer complications and provide a higher detection rate for diagnosis. In recent years, interest in the transperineal biopsy approach has been growing [14,15,16,17], as the benefit appears to be not only in terms of reduced complications but also in terms of cancer characterization [9].

In our retrospective analysis, the detection rate of any PCa on mpMRI-positive cases was similar to the one observed by others [8,14,15,16,17]. Overall, PCa was detected more significantly in biopsy-naïve patients than in patients undergoing prior biopsy, while negative biopsies were more frequent in prior biopsy.

The CDR between targeted and standard biopsy were not significantly different and in line with experiences already reported in the literature [8,9,10]. We did not observe an advantage of CDR by using only one biopsy technique; instead, the combination of the TBx and SBx approaches has shown a significant 9.6% added value in csPCa detection. Our results confirm previous studies showing that 13–16% of csPCa were missed by TBx in comparison with a combination of SBx and TBx [18,19]. The CDR of targeted biopsy has shown superior performance for detection of prostate cancer, but the role of systematic biopsy should not be overlooked, and the combination of targeted and systematic biopsy is essential [9]. Similarly, the combined TBx and SBx approach showed an added value of 10.3% and 9.9% in reducing the risk of any PCa missing after TBx or SBx alone, respectively. Obviously, the advantage of a reduced risk must be weighed with the corresponding increase in biopsy cores and the consequent increase in possible complications related to the biopsy. From this point of view, the transperineal approach has the advantage of a lower risk of sepsis than the transrectal route [8,9], thus representing a good alternative to conventional US-guided transrectal biopsy, which is the current gold standard [11].

Our study highlighted a significant number of negative or indolent tumor biopsies, which could be reduced by a diagnostic pathway that includes more effective risk indicators. Although this area has benefited from mpMRI, the problem of overdiagnosis and the excessive number of unnecessary biopsies is still debated. In addition to PSA and its derivatives, a large number of new blood and urinary biomarkers to assist in biopsy decisions have appeared in clinical trials, largely thanks to advances in genomic technologies, among which the Prostate cancer antigen 3 (PCA3) [20,21], the SelectMDx multiplex biomarker [22,23,24], the ExoDx prostate Intelliscore [25,26], the Prostarix Risk Score [27,28], and the Prostate-specific G-protein coupled receptor (PSGR) [29,30]. However, their contribution in predicting biopsy outcomes needs to be more rigorously weighed.

Our study is affected by several limitations, and primarily by its retrospective and non-randomized nature. The small size of our single study center in a heterogeneous study population is hampered by potential biases, among which the use of a single fusion-system, and a limited number of surgeons. Another factor that weakens the accuracy analysis is the variable number of cores (three to five) per lesion. Moreover, we do not have a quality review of mpMRI classification. These limitations could benefit from a multicentric, prospective, and randomized study using a combination of biomarkers for a more rigorous evaluation of a diagnostic pathway of csPCa that makes use of transperineal biopsy.

## 5. Conclusions

The transperineal TBx proved a detection rate of overall PCa and csPca consistent with the literature. The transperineal approach is feasible and can be performed under local anesthesia as conventional US-guided transrectal biopsy. All patients had no complication and no impact on erectile or urinary function. The combined TBx and SBx pathway in patients with a positive MRI is recommended to reduce the risk of csPCa missing. Future prospective larger-scale studies are needed to confirm our findings.

## Figures and Tables

**Table 1:** Characteristics of the participants. ^1^ IQR, Inter-Quartile Range.

Characteristics	All (*n* = 272)	Biopsy Naïve (*n* = 190)	Prior Negative Biopsy (*n* = 82)
Age (years), median (IQR ^1^)	68 (62–74)	68 (62–74)	68 (63–72)
PSA (ng/mL), median (IQR)	7.2 (4.8–10.1)	7.1 (4.8–9.6)	7.3 (4.9–11.7)
PIRADS *n*, (%)			
3	115 (42.3%)	73 (26.8%)	42 (15.4%)
4	129 (47.4%)	96 (35.3%)	33 (12.1%)
5	28 (10.3%)	21 (7.7%)	7 (2.6%)

**Table 2:** Overall cancer detection rate in naïve and prior biopsy cases. For each diagnosis, the number of cases and, in brackets, the percentage and 95% confidence interval are reported.

Cases	Biopsy Negative	csPCa (ISUP ≥ 2)	ciPCa (ISUP < 1)
Biopsy naïve 190/272 (69.9%)	99 (52.1%; 59.1–45.0)	60 (31.6%; 38.5–25.4)	31 (16.3%; 22.2–11.7)
Prior biopsy 82/272 (30.1%)	56 (68.3%; 77.4–57.6)	14 (17.1%; 26.6–10.4)	12 (14.6%; 23.9–8.6)

**Table 3:** Detection rate, diagnostic sensitivity, and specificity of TBx and SBx.

Cases	All *n* (%)	TBx *n* (%)	SBx *n* (%)	TBx/SBx *n* (%)
^1^ Biopsy negative	155 (57.0%)	183 (67.3%)	182 (66.9%)	155 (57.0%)
^1^ CDR for ciPCa	43 (15.8%)	28 (10.3%)	29 (10.7%)	43 (15.8%)
^1^ CDR for csPCa	74 (27.2%)	61 (22.4%)	61 (22.4%)	74 (27.2%)
^1^ False negative PCa		28 (10.3%)	27 (9.9%)	0 (0%)
^2^ Sensitivity for csPCa		82.4%	82.4%	100%
Specificity for csPCa		85.%	85.8%	78.3%

^1^ Percentage refers to the entire cohort (*n* = 272). ^2^ False negative includes biopsy negative plus ciPCa; since all diagnosed csPCa are derived from either TBx or SBx, the sensitivity of the combined TBx/SBx is 100%.

## Data Availability

The data presented in this study are available on request from the corresponding author.

## Supplementary Materials

The following are available online at https://www.mdpi.com/article/10.3390/cancers13194833/s1, Supplemental Methods: MRI Protocol and sequence parameters; Figure S1: Study flow chart; Figures S2–S4: Examples of mpMRI images of the study.
