# Supplementary material for: Multiparametric Magnetic Resonance Imaging-Ultrasound Fusion Transperineal Prostate Biopsy: Diagnostic Accuracy from a Single Center Retrospective Study
Source: Cancers (Basel). 2021 Sep 28;13(19):4833. doi: 10.3390/cancers13194833 (PMC8507636; doi:10.3390/cancers13194833)
Supplement: Supplementary file 1 [file cancers-13-04833-s001.zip › cancers-1309800-supplementary.pdf]

Supplemental Information

MRI Protocol and sequence parameters

The mpMRI was performed with a 1.5-T MRI scanner using a 32-channel pelvic phased-array coil combined with an endorectal coil (Philips Achieva). The protocol and the sequence parameters were in concordance with the current international prostate MRI guidelines (1) and were as follows:

- three orthogonal tri-planar T2-weighted (T2w) sequences (acquired planes = axial, typical repetition time= 3500 ms; minimum echo time= 93 ms; in-plane resolution= 0.27mm x 0.27 mm; slice thickness= 3 mm; acquired planes = coronal/sagittal, typical repetition time= 4000 ms; minimum echo time= 97 ms; in-plane resolution= 2.5mm x 2.4 mm; slice thickness= 3 mm);

- one axial diffusion-weighted imaging (DWI) sequence (typical repetition time= 5000 ms; minimum echo time= 75 ms; in-plane resolution= 0.7mm x 0.7 mm; slice thickness= 3 mm) with calculated high-b–value images (0, 50 and 1000 or 100, 600 and 1000 s/mm2), an ADC map;

- one dynamic contrast-enhanced sequence (DCE) with time resolution of 13 s (acquired planes = transverse, typical repetition time= 5 ms; minimum echo time= 1.8 ms; in-plane resolution= 0.7mm x 0.7 mm; slice thickness= 3 mm). Gadolinium contrast (Dotarem/Gadovist) was provided at an injection rate of 2-3 mls/s. The sequences used were T2w, DWI, DCE.

Areas of mpMRI that were suggestive of PCa were categorized by expert radiologists according to the PIRADS score, on a scale from 1 to 5, with higher numbers indicating a greater likelihood of csPCa (1); scores of 3 to 5 defined a positive mpMRI.

1. Weinreb, J.C.; Barentsz, J.O.; Choyke, P.L.; Cornud, F.; Haider, M.A.; Macura, K.J.; Margolis, D.; Schnall, M.D.; Shtern, F.; Tempany, C.M.; et al. PI-RADS Prostate Imaging - Reporting and Data System: 2015, Version 2. *European urology* **2016**, *69*, 16-40, doi:10.1016/j.eururo.2015.08.052.

Supplementary Figures

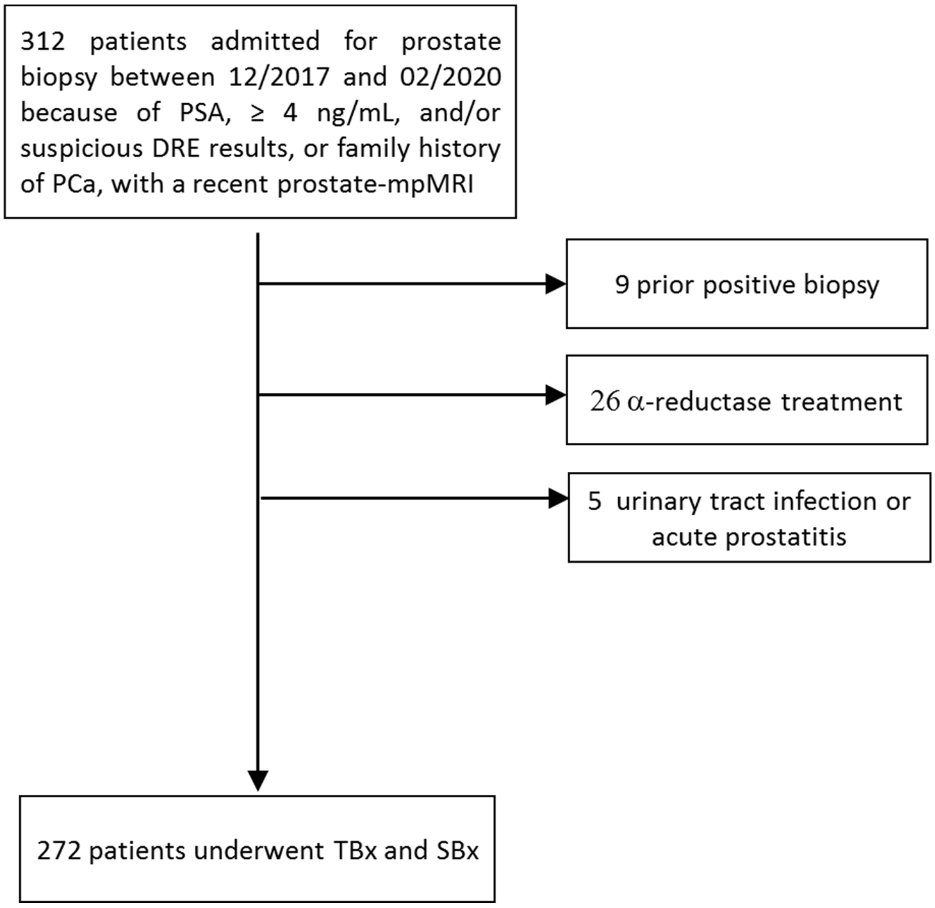

Figure S1. Study Flow chart showing inclusion/exclusion criteria.

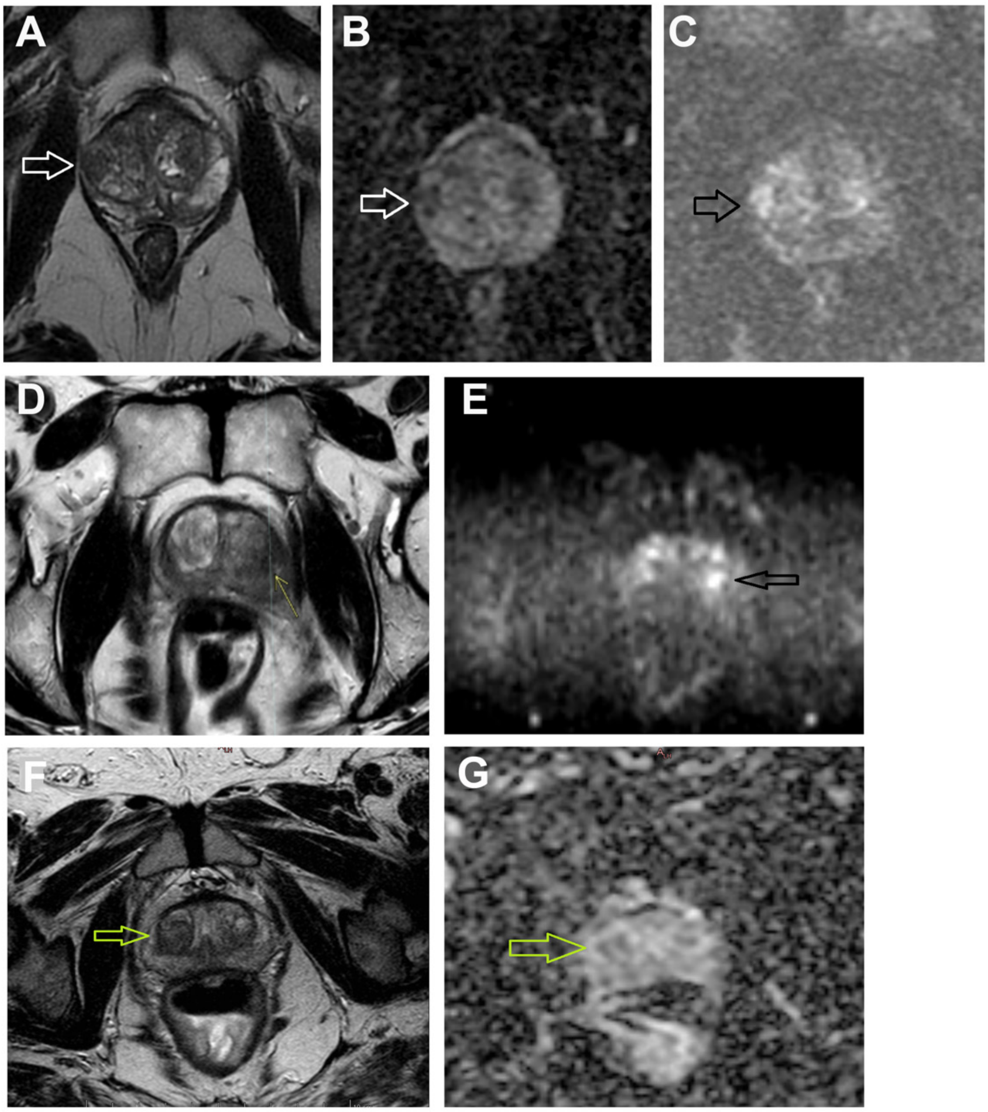

Figure S2. Examples of mpMRI with PIRADS 3 score.

(A-C) A 70-year-old patient with a serum PSA level of 6 ng/ml and fusion biopsy showing ISUP 1 in the random cores. Axial T2WI shows an area of decreased signal intensity in the right posterior transition zone (white arrow) with (B) ADC restricted diffusion (white arrows) with (C) high *b*-value DWI (1500 s/mm^2, black arrow).

(D-E). A 68-year-old patient with a serum PSA level of 8 ng/ml and fusion biopsy showing ISUP 1 in the random and fusion cores. Axial T2WI shows an area of decreased signal intensity in the left peripheral zone (yellow arrow) with (b) high b value DWI (2000 s/mm^2, black arrow).

(F-G) A 64-year-old patient with a serum PSA level of 11.68 ng/ml and fusion biopsy showing ISUP 3 in the random cores. (F) Axial T2WI shows an area of decreased signal intensity in the right posterior transition zone (arrow) with (G) ADC restricted diffusion (arrow).

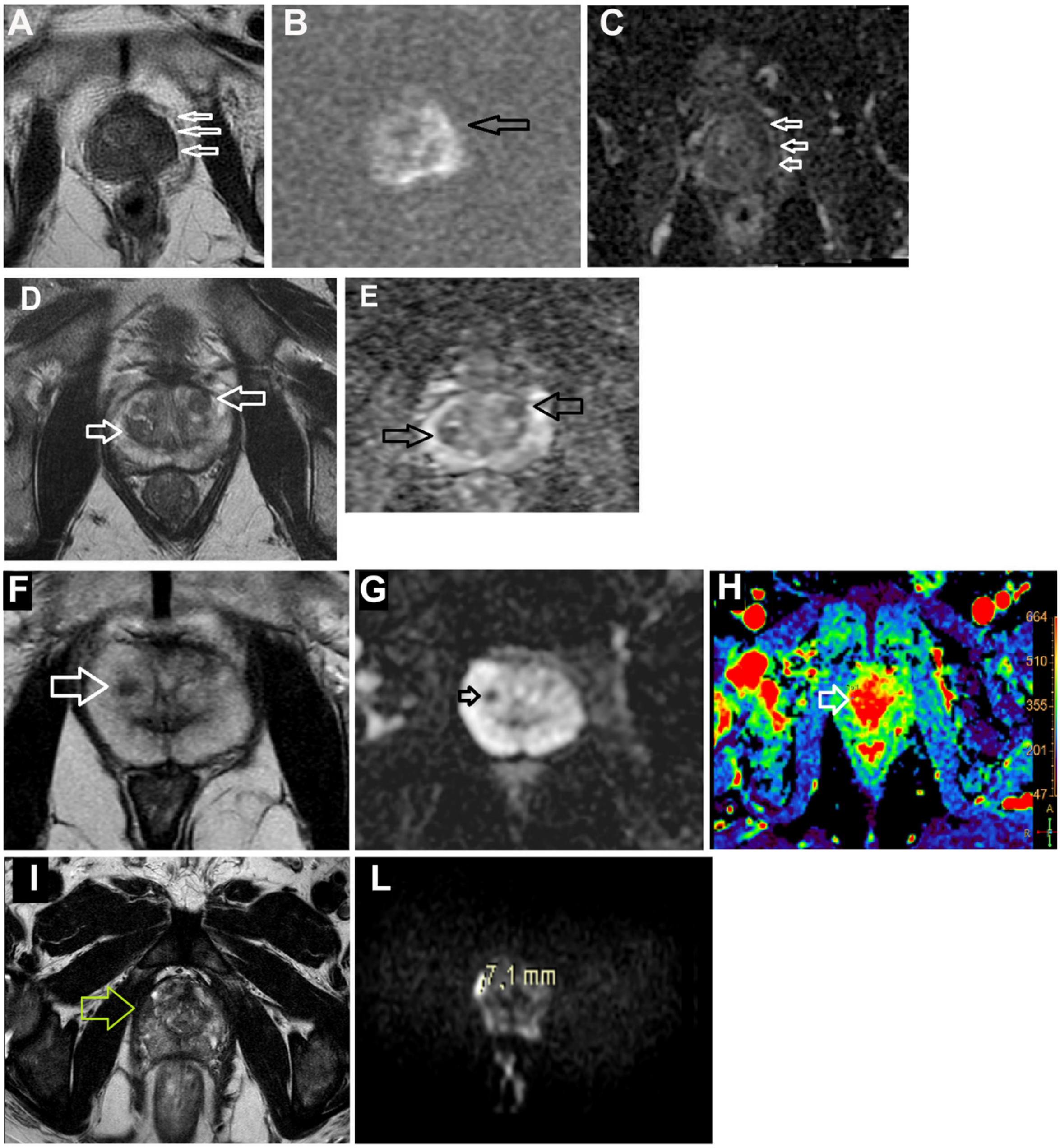

Figure S3. Examples of mpMRI with PIRADS 4 score. (A-C) A 68-year-old patient with a serum PSA level of 9 ng/ml and fusion biopsy showing ISUP 3 in the random cores. (A) Axial T2WI shows a 13 mm area of decreased signal intensity in the left anterior transition zone (white arrows) with (B) high *b*-value DWI (1500 s/mm^2, black arrows) and (C) ADC restricted diffusion (white arrows). (D-E) A 63-year-old patient with a serum PSA level of 4.44 ng/ml and fusion biopsy showing ISUP 1 in the random and fusion cores. (D) Axial T2WI shows 2 areas of decreased signal intensity in the left anterior and in the right posterior transition zone (white arrows) with (E) ADC restricted diffusion (black arrows). (F-H) A 63-year-old patient with a serum PSA level of 10.6 ng/ml and fusion biopsy showing ISUP 3 in the random cores. (F) Decreased signal in axial T2WI (white arrow) and (G) axial ADC map demonstrates restricted diffusion (black arrow). (H) Color perfusion map created using postprocessing software for dynamic contrast enhanced MR imaging acquisition demonstrates suspicious perfusion kinetics for prostate cancer. (I-L) A 70-year-old patient with a serum PSA level of 15 ng/ml and fusion biopsy showing ISUP 3 in the fusion cores. (D) Axial T2WI shows an area of decreased signal intensity in the right peripheral zone (arrows) with (E) high *b*-value DWI (1500 s/mm^2).

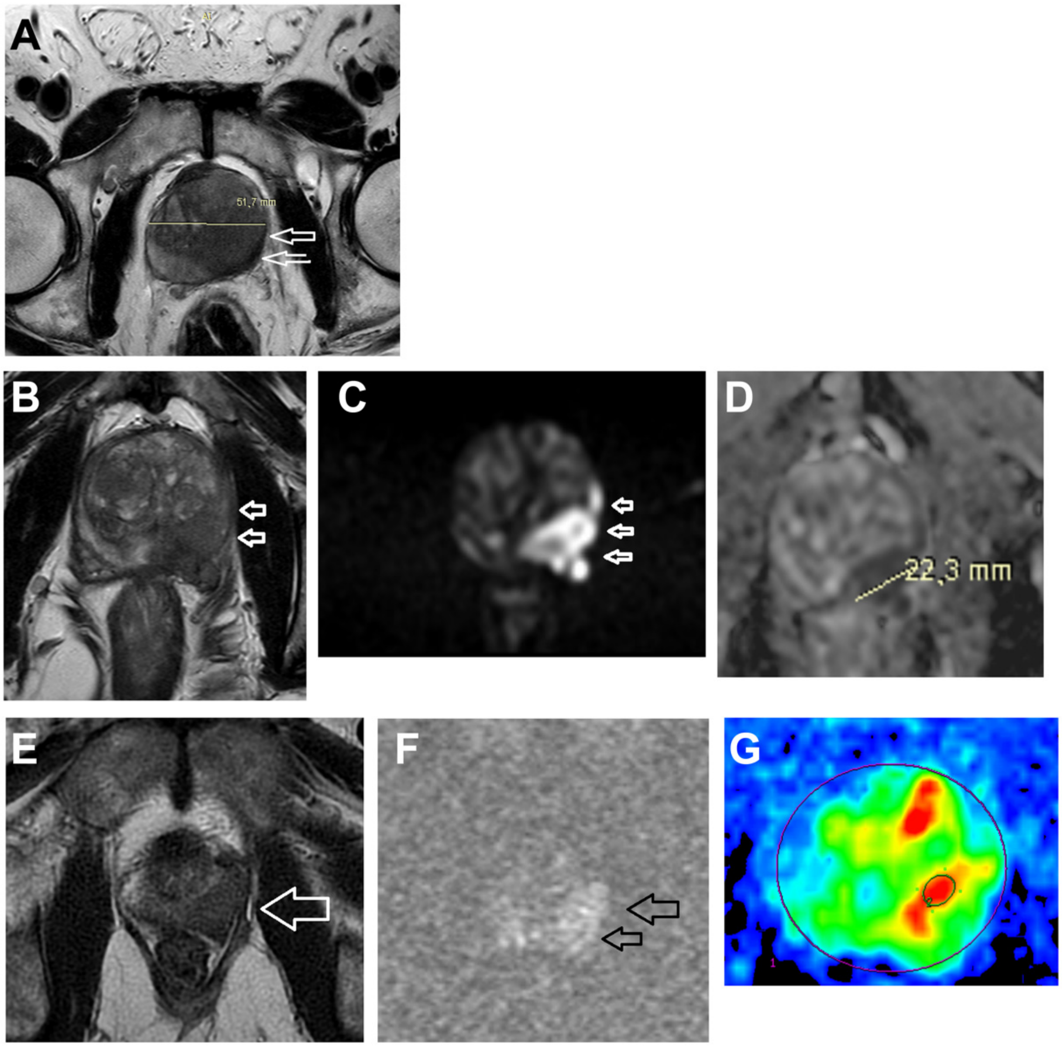

Figure S4. Examples of mpMRI with PIRADS 5 score.

(A) A 68-year-old patient with a serum PSA level of 9.36 ng/ml and fusion biopsy showing ISUP 4 both in the random and fusion cores. Axial T2WI shows a large region of decreased T2 signal with poorly defined margins in left in the mid lateral peripheral zone (white arrows) with suspicious extra-prostatic extension.

(B-D) A 69-year-old patient with a serum PSA level of 11.5 ng/ml, with suspicious DRE of the left gland and fusion biopsy showing ISUP 3 in the fusion cores. (B) Axial T2WI shows a 22 mm area of decreased signal intensity in the left peripheral zone (white arrows) with capsular contact and seminal vesicles invasion and (C) high *b*-value DWI (2200 s/mm^2, white arrows) and (D) ADC restricted diffusion.

(E-F) A 65-year-old patient with a serum PSA level of 8.23 ng/ml and fusion biopsy showing ISUP 4 both in the random and fusion cores. Axial T2WI shows a large region of decreased T2 signal in the left peripheral zone (white arrows) with (E) high *b*-value DWI (1500 s/mm^2, black arrows) (F) Color perfusion map created using postprocessing software for dynamic contrast enhanced MR imaging acquisition demonstrates suspicious perfusion kinetics for PCa.
